# Renal vein thrombosis mimicking urinary calculus: a dilemma of diagnosis

**DOI:** 10.1186/s12894-015-0054-1

**Published:** 2015-07-02

**Authors:** Yimin Wang, Shanwen Chen, Wei Wang, Jianyong Liu, Baiye Jin

**Affiliations:** Department of Urology, the First Affiliated Hospital of Medical College, Zhejiang University, No. 79 Qing Chun Road, 310003 Hangzhou, China; Sidney kimmel Comprehensive Cancer Center, Johns Hopkins University School of Medicine, 21128 Baltimore, USA

**Keywords:** Renal calculus, Diagnosis, Renal vein thrombosis, Computed tomography

## Abstract

**Background:**

Renal vein thrombosis (RVT) with flank pain, and hematuria, is often mistaken with renal colic originating from ureteric or renal calculus. Especially in young and otherwise healthy patients, clinicians are easily misled by clinical presentation and calcified RVT.

**Case presentation:**

A 38-year-old woman presented with flank pain and hematuria suggestive of renal calculus on ultrasound. She underwent extracorporeal shock wave lithotripsy that failed, leading to the recommendation that percutaneous lithotomy was necessary to remove the renal calculus. In preoperative view of the unusual shape of the calculus without hydronephrosis, noncontrast computed tomography was taken and demonstrated left ureteric calculus. However computed tomography angiography revealed, to our surprise, a calcified RVT that was initially thought to be a urinary calculus.

**Conclusion:**

This case shows that a calcified RVT might mimic a urinary calculus on conventional ultrasonography and ureteric calculus on noncontrast computed tomography. Subsequent computed tomography angiography disclosed that a calcified RVT caused the imaging findings, thus creating a potentially dangerous clinical pitfall. Hence, it is suggested that the possibility of a RVT needs to be considered in the differential diagnosis whenever one detects an uncommon shape for a urinary calculus.

## Background

The clinical manifestations of renal vein thrombosis (RVT) in adult patients vary, along with the rapidity and degree of venous occlusion. Symptoms of RVT can include acute flank pain, hematuria, and deterioration of renal function, although RVT is usually asymptomatic. RVT with flank pain and hematuria is often misdiagnosed as renal colic originating from a ureteric or renal calculus, especially in young and otherwise healthy patients. This report describes an unusual patient with RVT, who presented with flank pain and calcified RVT mimicking a urinary calculus.

## Case presentation

A 38-year-old woman presented to the emergency room in a regional hospital with nausea and left flank pain that had suddenly started 12 h earlier. She had a history of left flank trauma, having fallen from 2-meter- height 5 years earlier. Physical examination revealed no tenderness over the abdomen or flank and normal chest auscultation. Blood analysis was within normal limits, except for leukocytosis (12.7 × 10^9^/L). Routine urine analysis showed microscopic hematuria without white blood cells or proteins. Abdominal ultrasonography showed an enlarged kidney and a 16-mm hyperechoic focus in the left renal pelvis, suggesting a diagnosis of renal calculus. The patient was therefore administered extracorporeal shock wave lithotripsy at the regional hospital. A follow-up ultrasound examination 2 weeks later showed that the shape and size of the hyperechoic focus had not changed.

**Fig.1:**
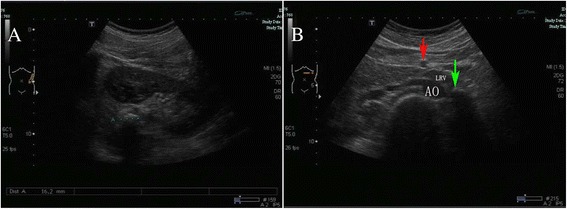
The images from the ultrasonography. **a** An abdominal ultrasonography demonstrated 16-mm hyperechoic, echogenic focus in the left renal pelvis. **b** A color Doppler ultrasonography indicated the hyperechoic focus (*green arrow*) in the left renal vein (LRV). Aorta (AO); left renal vein (LRV); superior mesenteric artery (*red arrow*)

**Fig. 2:**
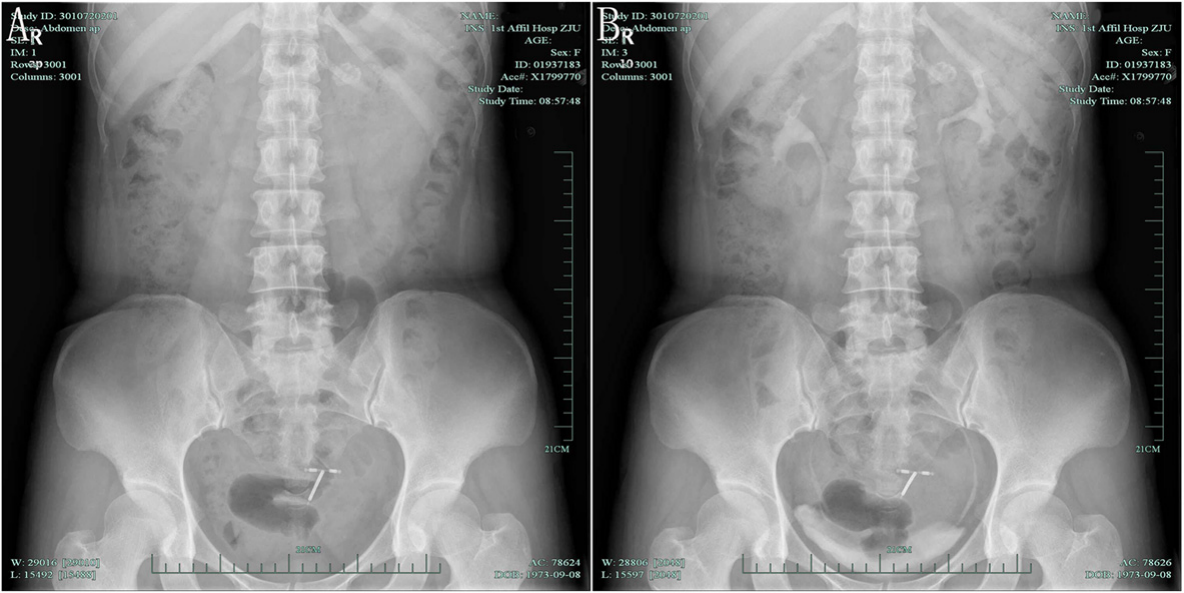
The images from the abdominal plain film and intravenous pyelography. **a** An abdominal plain film showed a left-sided renal calcification. **b** After injecting contrast medium, intravenous pyelography showed normal renal pelvis and a patchy shadow within the left kidney contour

Due to left lower back pain and the persistent left hyperechoic focus in the renal area, the patient was referred to our hospital for further evaluation and treatment. Urine analysis and ultrasound examination showed the same results as in the regional hospital (Fig. 1a). An abdominal plain film showed a left-sided renal calcification (Fig. 2a), but there was no evidence of a palpable tender mass or an audible abdominal bruit. Laboratory data, except for urinalysis results, were within normal limits. Because of the failure of extracorporeal shock wave lithotripsy, percutaneous lithotomy was recommended.

**Fig. 3:**
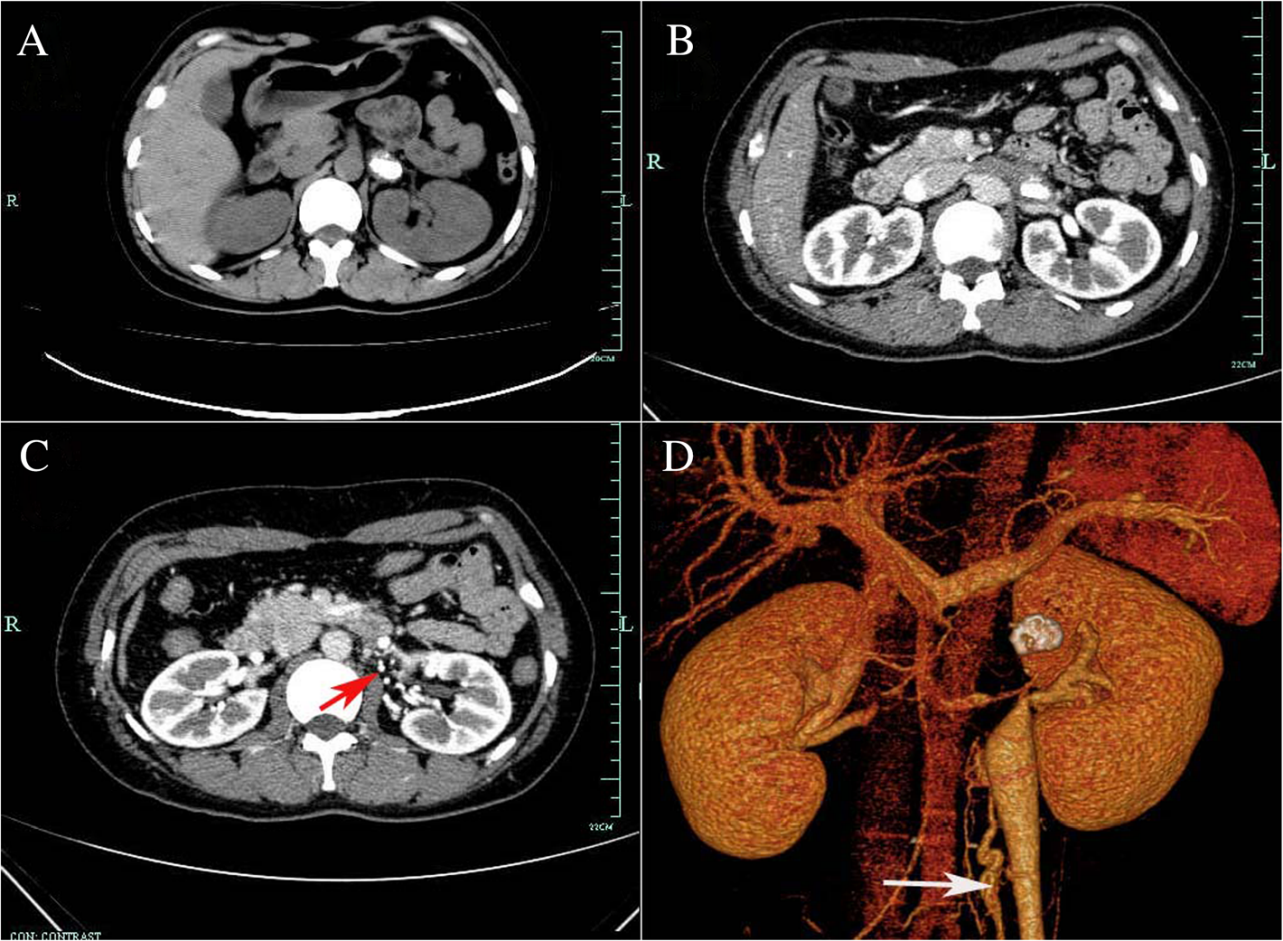
The images from the computed tomography (CT). **a** The noncontrast CT demonstrated left ureter calculus. **b** The contrast enhanced CT indicated a hyperdense mass in the left renal vein. **c** The contrast enhanced CT indicated peripheral veins (*red arrow*) around the left renal hilum. **d** Three-dimensional CT clearly displayed the calcified RVT with varicose ovary vein (*white arrow*)

Because the shape of the calculus was unusual and there was no evidence of hydronephrosis, the diagnosis of calculus was questioned. Further evaluation to determine the true nature of the hyperechoic lesion included computed tomography (CT) without contrast, which showed the calculus in the left ureter (Fig. 3a). In addition, intravenous pyelography showed that the pelvic areas around both kidneys were normal, although a patchy shadow was observed above the left renal pelvis (Fig. 2b). Color Doppler ultrasonography showed the hyperechoic focus in the left renal vein (Fig. 1b). CT angiography indicated a hyperdense mass in the left renal vein, suggesting a calcified thrombus, as well as occlusion of the left renal vein, varicosity in the left ovarian vein, and peripheral veins around the left renal hilum (Fig. 3b–d).

The patient was actively monitored. Three months later, there was satisfactory regression of microscopic hematuria and left lower back pain. After 15 months, the patient remained asymptomatic. Routine urine analysis, serum creatinine and glomerular filtration rate were normal. Periodic ultrasound examination also revealed no morphologic changes in the affected kidney, with RVT remaining in situ.

## Discussion

RVT is defined as thrombus formation in the main and/or branch renal veins. This may result in full or partial blockage of renal veins and, subsequently, to a series of pathological changes and clinical manifestations [1–3]. RVT is the most frequent vascular abnormality in newborns. In most infants, RVT is bilateral and is accompanied by dehydration after diarrhea or vomiting [4]. RVT is rarely observed in healthy adults; in most affected adults, it is unilateral and may be accompanied in 15–20 % of patients by nephrotic syndrome. RVT is associated with abdominal surgery, including laparoscopic cholecystectomy, trauma, tumor invasion of the renal vein or invasion by primary retroperitoneal diseases.

The pathophysiology of venous thrombosis has been reported to involve a combination of three interrelated factors: endothelial damage, stasis, and hypercoagulability. Although a single abnormality may precipitate thrombosis, most venous thromboses are triggered by at least two of these factors. The causes and mechanisms of RVT are no different from venous thromboses elsewhere in the body (Table 1).

**Table 1:** Causes of renal vein thrombosis

Endothelial damage [18, 19]	Stasis [18, 20]	Hypercoagulability [18, 21]
Blunt trauma	Severe volume losses e.g., GI fluid loss, haemorrhage, dehydration	Nephrotic Syndrome: Membranous glomerulonephritis, Membranoproliferative glomerulonephritis, Focal segmental glomerulosclerosis, Minimal change disease
Trauma during venography	Post transplant distortion/kink of renal vein	Sepsis: Generalized/Localized (in and around kidney)
Renal transplant	Primary retroperitoneal processes with renal vein compression	Puerperium
Infiltration by tumour	Severe volume losses e.g., GI fluid loss, haemorrhage, dehydration	Disseminated malignancy
Acute rejection		Oral contraceptives
Vasculitis		Puerperium
Spontaneous micro-trauma to the endothelium e.g., in homocystinuria		Intrinsic Hypercoagulability: Factor V Leiden (Resistance to activated protein C), Prothrombin gene mutation (G20210A), Deficiency of Protein S, Deficiency of Protein C, Deficiency of anti-thrombin, Unknown/Poorly Understood causes, Anti-phospholipid Syndrome, Primary & Secondary e.g., SLE, Behcet’s disease, AIDS-associated nephropathy

RVT often commences in small intrarenal veins and subsequently extends via larger interlobar veins to the main renal veins and even to the inferior vena cava, where it may cause pulmonary embolism [5]. The clinical presentation of RVT in adults depends on the rate, extent, and completeness of thrombus formation. Patients may be asymptomatic, have minor nonspecific symptoms such as nausea and weakness, or have more major nonspecific symptoms such as upper abdominal pain, flank pain, and hematuria [6]. RVT is likely underreported, as some patients may go undiagnosed due to a lack of clinical manifestations. Establishing the diagnosis is essential because of the possible sequelae, including pulmonary embolism and progressive renal impairment related to vascular compromise, and the risks of potentially harmful treatment (anticoagulation or thrombolysis). In young patients, flank pain and hematuria are usually regarded as symptoms of renal and ureteric calculi; and similar clinical presentation due to other causes is often overlooked in the emergency room. Pulmonary thrombosis may occur in as many as 50 % of patients with RVT, and RVT complicated by pulmonary embolism can have similar symptoms, suggesting a high index of differential diagnosis not to miss the diagnosis of RVT [7].

In the absence of specific diagnostic laboratory tests and the paucity of clinical manifestations, imaging remains the cornerstone of diagnosing RVT. The gold standard method for diagnosing RVT is selective renal venography, but this is not often performed because of the invasiveness of this procedure, including exposure to high levels of radiation, injection of iodinated contrast, and the potential risk of venous injury causing de novo RVT [8].

In diagnosing RVT, ultrasound imaging and Doppler ultrasonography are not recommended because their results are inconsistent and operator-dependent. Ultrasound scans may show an enlarged kidney, and a hyper-echogenic kidney is observed in approximately 90 % of patients during the early phase of acute RVT [9]. Color Doppler ultrasound is ineffective in detecting segmental venous thrombosis, but is superior to conventional ultrasound and abdominal plain film in detecting flow in the renal artery and vein. Although color Doppler ultrasound is highly sensitive when performed by an experienced operator, but remains highly operator-dependent [8]. Rarely, the calcified vessel walls of the renal venous branches coursing through the sinus may be mistaken for a renal calculus on ultrasonography. In the patient described here, there was no evidence of turbulent flow within the calcified RVT. Thus, RVT was not considered in the initial evaluation, although subsequent color Doppler yielded results suggestive of RVT.

CT is currently the imaging method of choice for diagnosing RVT, as it is non-invasive, is somewhat less expensive than other methods, can be performed quickly, and has a high diagnostic accuracy. CT scans have shown high sensitivity (92 %) and specificity (100 %) in diagnosing these lesions and is therefore recommended as an initial diagnostic tool [3]. Our findings showed that a renal calcified RVT may mimic a ureter calculus on noncontrast CT scans, with subsequent CT angiography used in the definitive diagnosis of a calcified RVT. CT angiography has shown nearly 100 % sensitivity in diagnosing RVT [2]. The diagnostic accuracy of CT angiography is similar to that of renal venography, with CT angiography having additional benefits, being a rapid, cost-effective, non-invasive method for evaluating the renal vasculature and for detecting renal tumors and other renal pathologies simultaneously. The disadvantages of CT include exposure to radiation and use of nephrotoxic iodinated contrast media, a potential risk factor in patients with impaired renal function [10].

The treatment modalities for patients with RVT have changed over the past decades, from surgical to predominantly medical management, consisting of initial intravenous and subsequent oral anticoagulation [11]. Asymptomatic patients with unilateral RVT may not require any specific treatment [12]. Rather, active surveillance, along with supportive measures including salt and protein restriction, may partially reverse the hypercoagulability, as in this patient. However, if a patient’s condition deteriorates due to either the progression of thrombosis or embolism, active intervention should be considered.

RVT may be diagnosed incorrectly as renal colic or renal cell carcinoma on abdominal ultrasonography [12–14]. Results in our patients showed that a calcified RVT may mimic a urinary calculus on conventional ultrasonography, abdominal plain film and noncontrast CT. Renal stones may also resemble paragonimus calcified ova [15], renal artery aneurysms [16] and acute renal infarctions [17]. Thus, awareness of the conditions that could mimic those observed during the generation of a urinary calculus is important, particularly if a percutaneous procedure is considered. Ultrasonography alone is not sufficient to rule out RVT in these patients, suggesting the need for CT angiography in evaluating our patients.

## Conclusions

Results in this patient showed that a calcified RVT might mimic a urinary calculus on conventional ultrasonography, abdominal plain film and noncontrast CT. Subsequent CT angiography revealed a calcified RVT, changing the course of diagnosis, treatment and follow-up. Examination of an unusual or uncommon shape for a calcified mass suggests the need to include a calcified RVT in the differential diagnosis.

### Consent

Written informed consent was obtained from the patient for publication of this manuscript and accompanying images. A copy of the written consent is available for review by the Editor-in-Chief of this journal.

## Abbreviations

RVT: Renal vein thrombosis
CT: Computed tomography
